# Emerging oral *Treponema* membrane proteins disorder neutrophil phosphoinositide signaling via phosphatidylinositol-4-phosphate 5-kinase

**DOI:** 10.3389/froh.2025.1568983

**Published:** 2025-04-03

**Authors:** Natalie K. Anselmi, Stephen T. Vanyo, Michelle B. Visser

**Affiliations:** Department of Oral Biology, The State University of New York at Buffalo, Buffalo, NY, United States

**Keywords:** periodontitis, neutrophil, *Treponema*, phosphoinositide, actin

## Abstract

**Background:**

Periodontitis (PD) is a group of inflammatory pathologies characterized by destruction of the tooth-supporting tissues. During PD, dysbiosis of the oral biofilm disrupts the host immune response and supports growth of pathogenic bacteria including the spirochetes *Treponema denticola* (*Td*), *T. maltophilum* (*Tm*), and *T. lecithinolyticum* (*Tl*)*.* The outer membrane protein of *Td*, Msp, perturbs the function of neutrophils by modulating phosphoinositide (PIP) signaling. While *Tm* and *Tl* have similar outer membrane proteins, MspA and MspTL respectively, little is known of how these proteins affect neutrophil function.

**Methods:**

This study examines putative mechanisms by which *T. maltophilum* MspA and *T. lecithinolyticum* MspTL inhibit neutrophil chemotaxis. Murine bone marrow neutrophils were treated with recombinant MspA or MspTL protein. Protein phosphorylation was assessed via immunoblot, phosphate release by malachite green assay, and PTEN and SHIP phosphatase activity through immunoprecipitation, enzymatic assays, and chemical inhibition. PIP quantification was assessed by immunofluorescence microscopy and Mass ELISAs, while small GTPase activity was measured with G-Protein Activation Assays. Neutrophil F-actin localization was determined through immunofluorescence.

**Results:**

MspA and MspTL increase phosphate release in neutrophils, but unlike Msp, they do not affect PTEN or SHIP activity, despite modulating cellular levels of multiple PIP species [PI(3,4)P_2_, PI(4,5)P_2_, and PIP_3_]. Overall, MspA and MspTL differentially affected the metabolism of individual PIP species, but both increased PI(4,5)P_2_ levels in a PIP5K-dependent manner. Downstream effects of disrupted PIP signaling included inhibition of Akt and Rac1 activation and increased cortical F-actin localization.

**Conclusions:**

Understanding distinct mechanistic relationships between novel Msp proteins and neutrophils provides important insight into how these understudied bacteria promote periodontitis progression.

## 1 Introduction

Periodontal disease represents a spectrum of chronic inflammatory pathologies affecting more than 40% of adults 30 years and older and 70% of adults 65 years and older in the United States. Periodontal disease ranges from gingivitis, which is characterized by reversible inflammation confined to the gum tissue, to a range of categories of periodontitis (PD). PD is characterized by progressive destruction of the periodontium, manifested by irreversible loss of tooth-supporting soft tissue and underlying alveolar bone, and is the leading cause of tooth loss worldwide (1, 2). PD is initiated by disrupted homeostasis of the complex oral microbiota and the immune system, driven by a transition to an inflammogenic community (3–5).

Oral spirochetes proliferate to high abundance in the dysbiotic subgingival biofilm during PD and preferentially colonize the deepest part of diseased periodontal pockets (6–9), in close association with immune cells at the tissue interface, including neutrophils (10, 11). *Treponema denticola* is the most well-characterized oral spirochete and classically colocalizes with *Porphyromonas gingivalis* and *Tannerella forsythia* (classically referred to as the “red complex”) during severe PD (12, 13). There are ten named oral *Treponema* species, including *T. maltophilum* and *T. lecithinolyticum* which are considered important emerging “core periodontitis” pathogens (10, 11, 14, 15), yet are comparatively understudied. Numerous studies have demonstrated the abundance and prevalence of these species in deep periodontal pockets during different forms of periodontitis (11, 14, 16–18) and infected root canals associated with apical periodontitis or secondary failure (19–21). *T. maltophilum* and *T. lecithinolyticum* have been reported to be more prevalent in treatment-resistant endodontic lesions (22) and less prevalent in periodontitis “resistant” individuals (>65 years of age, 20 or more teeth, no periodontitis history) (11). Despite their prevalence, little remains known regarding the pathogenic potential of these *Treponema* species and mechanistically how they interact with immune cells. The study of oral spirochetes remains limited due to their unique fastidious nutritional requirements, complex growth media required for *in vitro* culture (23) and limited tools available for genetic manipulation (24, 25). Currently, limited *T. maltophilum* and *T. lecithinolyticum* isolates and complete genomic information are readily available. While development of tools for genetic mutagenesis and complementation studies in *T. denticola* continue to advance (25–28), none are currently available for *T. maltophilum* or *T. lecithinolyticum*.

The major outer sheath protein (Msp) of *T. denticola* is a highly expressed and immunogenic virulence factor, eliciting strong antibody responses in human subjects (29, 30)*.* Msp forms a trimeric pore complex with dual adhesin and porin functions in the outer membrane of *T. denticola* and secreted outer membrane vesicles (30–33). *T. denticola* has a broad pathogenic capacity toward host cells (reviewed in references (34, 35). Msp stimulates the expression of host proteases such as matrix metalloproteinases, promoting tissue destruction and bone reabsorption (35), has hemagglutination and hemolytic activity towards erythrocytes (36, 37), and disrupts calcium-ion-mediated cell signaling and actin uncapping in fibroblasts (38, 39) and chemotaxis in neutrophils (40, 41). Furthermore, *T. denticola* lacking Msp increases neutrophil migration *in vitro* (40), confirming the importance of this protein in intact treponemes to impair neutrophil function.

Msp-like surface proteins have been identified in *T. maltophilum* (MspA) and *T. lecithinolyticum* (MspTL) (42, 43). *T. maltophilum* and *T. lecithinolyticum* are more closely phylogenetically related to each other than either is to *T. denticola.* Correspondingly*,* MspA and MspTL are most similar to each other and distinct from Msp (14, 16, 17, 44). Early research showed that MspTL is a major membrane-associated component of the *T. lecithinolyticum* outer membrane (43) and that MspA in *T. maltophilum* forms a heat-modifiable, detergent and trypsin-stable high molecular-mass membrane-associated protein complex (42), similar to Msp in *T. denticola* (32, 45). We recently demonstrated computationally that MspA and MspTL are predicted to form large β-barrel monomers composed of 20 all-next-neighbor antiparallel β strands, likely to adopt a homotrimer formation, and experimentally validated amphiphilic integral membrane-association, oligomerization and surface accessibility for both native protein complexes (46). Limited functional analysis of MspA or MspTL biological activity towards host cells has been performed. We have recently shown *in vitro* that purified recombinant MspA or MspTL monomer proteins can impair murine neutrophil chemotaxis and that surface expression of MspA or MspTL in an *E. coli* surrogate system delays chemotaxis in a murine air pouch model of inflammation (46). MspTL and MspA can also induce the release of pro-inflammatory interleukins such as IL-1, IL6, and IL-8 from human monocytes and fibroblasts (47) and expression of ICAM-I (44), while MspTL has been found to increase human monocyte adhesion to microvascular endothelial cells (47).

Neutrophils are a major innate immune cell in the oral cavity crucial for maintaining gingival health. They comprise the majority of innate immune cells recruited to the gingival tissue and crevice and are positively correlated with PD severity (48–50). Furthermore, a lack of neutrophil infiltration into the oral cavity, congenital defects in neutrophil development, or defects of neutrophil function lead to an increased severity of PD (51–54). Neutrophils are dynamic cells that perform diverse biological functions including directed migration, extravasation into tissue sites, and antimicrobial actions. Effective neutrophil functionality is tightly regulated by complex phosphoinositide-associated signaling pathways (55–57).

Phosphoinositides (PIPs) are a class of signaling lipids, whose inositol head group can be phosphorylated by lipid kinases on the third, fourth, or fifth position to elicit different signaling functions (Supplementary Figure 1A) (55, 58). Among these enzymes is phosphoinositide 3-kinase (PI3K), which generates the second messenger phosphatidylinositol(3,4,5)trisphosphate (PIP_3_) by phosphorylating the D3 position of the inositol ring of phosphatidylinositol(4,5)bisphosphate [PI(4,5)P_2_], an integral component of the inner leaflet of the plasma membrane (PM). PIP_3_ recruits and activates numerous effectors at the PM to regulate neutrophil function. Appropriate temporal and spatial localization of PIP_3_ at the leading edge is an integral step to direct neutrophil chemotaxis (59, 60). In opposition to PI3K are phosphatases that hydrolyze PIP_3_: phosphatase and tensin homolog (PTEN) and SH2 domain-containing inositol 5′-phosphatase (SHIP). PTEN dephosphorylates PIP_3_ to PI(4,5)P_2_ while the hematopoietic and osteolineage-restricted SHIP1 and ubiquitously expressed SHIP2 dephosphorylate PIP_3_ to form phosphatidylinositol(3,4)bisphosphate [PI(3,4)P_2_], a lipid second messenger that shares some effectors with PIP_3_ (Supplementary Figure 1B) (61, 62). Downstream effectors of PIP_3_ include Akt and Rac1, which play complex interconnected roles in regulating neutrophil behaviors including chemotaxis (63, 64). Mechanistically, Msp disrupts the balance of phosphoinositide signaling by activating PTEN and inhibiting PI3K activity leading to inhibition of downstream Rac1 and Akt signaling to impair neutrophil function (33, 40, 41, 65–67).

*T. maltophilum* MspA and *T. lecithinolyticum* MspTL have recently been shown to modulate neutrophil function (46), but knowledge of their molecular processes remains limited. Here we focus on examining how these virulence factors modulate the production of PIP species and the activity of associated signaling pathways. Awareness of the novel mechanisms these understudied bacteria manipulate to impact neutrophil function is crucial for ongoing therapeutic development for PD to improve oral health.

## 2 Materials and methods

### 2.1 Expression and purification of recombinant MspA and MspTL

*T. maltophilum* ATCC51939 *mspA* codon-optimized sequence (lacking the 19 amino acid signal peptide sequence) for *E. coli* expression was prepared by GeneScript, enzyme digested and cloned in frame with the N-terminal His-tag at the NdeI/HindIII enzyme sites of pET30a to form protein expression construct pET30a-Msp. *T. lecithinolyticum* ATCC 700332 *mspTL* (lacking the 19 amino acid signal peptide sequence) in pQE30 was used for recombinant MspTL isolation (44). *E. coli* BL21 Star (DE3) (ThermoFisher) containing pET30a-MspA or *E. coli* M15 containing pQE30-MspTL were grown from an overnight culture to an OD_600_ of ∼0.6 in 500 ml LB medium containing appropriate antibiotics (*E. coli* BL21-pET30 constructs were grown with 100 μg/ml ampicillin. *E. coli* M15 contains a pREP4 plasmid that confers kanamycin resistance, and so *E.coli* M15-pQE30 constructs were grown with 100 μg/ml ampicillin and 25 μg/ml kanamycin.) in a 2 L flask at 37°C with shaking (200 RPM). Isopropyl-β-d-thiogalactopyranoside (IPTG, final concentration 1 mM) was added, and the culture was incubated for 4 h at 37°C with shaking to induce protein expression. Bacteria were harvested by centrifugation (2,000 × *g* for 40 min at 4°C).

Recombinant His-tagged Msp lacking the signal peptide from *T. denticola* 35405 was isolated as previously described (40). Recombinant His-tagged MspA and MspTL proteins were purified by gravity flow. Cell pellets were suspended in binding/washing (B/W) buffer (20 mM NaH_2_PO_4_; 500 mM NaCl; 20 mM imidazole, pH 7.4) containing Protease Inhibitor Cocktail (1% v/v; Sigma) and 0.5 μg/ml of Pierce TM Universal Nuclease for Cell Lysis (Thermo Fisher Scientific), and lysed using an EmulsiFlex®-C3 high-pressure homogenizer (1,000–1,500 psi at 4°C, three 30 s pulses). Soluble and insoluble fractions were separated by centrifugation (15,500 × *g* for 30 min at 4°C) and the insoluble fraction was solubilized in 10 ml of urea binding buffer (8 M Urea, 300 mM NaCl, 50 mM NaH_2_PO_4_, 10 mM imidazole, 1 mM THP; pH 8.0) for 60 min at room temperature and then centrifuged (10,000 × *g* for 30 min at 4°C) to remove cellular debris. Poly-Prep Chromatography Columns (Bio-Rad) were loaded with 2 ml of His-Bind Resin (Millipore) to yield a 1 ml resin bed volume. The resin bed was washed with 3 column volumes of ultrapure diH_2_O, charged with 5 column volumes of nickel buffer (100 mM NiSO_4_ in diH_2_O), and equilibrated with urea binding buffer before the supernatant was added. After allowing the supernatant to flow through, the resin was washed with 10 column volumes of urea binding buffer and the recombinant proteins were eluted with 3 ml of urea elution buffer (8 M urea, 500 mM NaCl, 250 mM imidazole, 20 mM NaH_2_PO_4_; pH 8.0). Eluate was dialyzed sequentially against Buffer A (4 M urea, 20 mM ethanolamine; pH 11.7), 1:1 Buffer A, and Buffer B (2 M urea, 20 mM ethanolamine, 2 mM cystine; pH 11.7), Buffer C (20 mM Tris Base, 2 mM cysteine, 0.2 mM cystine; pH 10.7), and finally into PBS (pH 7.4). All buffers were made in dH_2_O and dialysis steps were performed at 4°C for 8 h. Protein concentrations were determined by BCA assay (Pierce) and integrity and purity were assessed by SDS-PAGE. Proteins were stored in PBS at −80°C.

### 2.2 Murine neutrophil isolation

Neutrophils were isolated from the bone marrow of mice using a Percoll density gradient (68). Briefly, C57BL/6J wild-type mice (male and female, 6 weeks old) were purchased from Jackson Laboratory (Bar Harbor, ME). Following exposure to CO_2_ and cervical dislocation, femurs and tibias were removed, and cells were isolated from bone marrow by fractionation into discontinuous Percoll (Sigma) gradients (80%, 65%, 55%). Mature neutrophils were isolated from the 80%–65% interface and red blood cells were lysed with RBC Lysis Buffer (BioLegend). Cells were manually counted using a hemacytometer and then used in assays. The University at Buffalo Institutional Animal Care and Use Committee approved all procedures.

### 2.3 Neutrophil coincubation treatment

*N*-formyl-methionine-leucine-phenylalanine (fMLP) is a bacterial peptide product known to stimulate neutrophils and was used as an activation stimulus throughout this study. Neutrophils (typically 1 × 10^6^ cells per condition unless noted) were incubated with either 100 nM rMsp, rMspA, or rMspTL alone for 30 min at room temperature or recombinant proteins followed by a 1 min exposure to 1 μM fMLP, as indicated, in HBSS++. Neutrophils exposed to HBSS++ were used as negative controls while positive controls consisted of cells stimulated with fMLP alone. Previous studies from our group support the use of these protein concentrations and incubation times, as these variables are within the range documented to produce observable effects on host cells (40, 67, 69).

### 2.4 Assessment of protein phosphorylation by immunoblotting

Neutrophils were treated with recombinant protein and fMLP as described above, lysed with 30 µl 4X SDS sample buffer, and boiled for 10 min. For immunoblot analysis, equal volumes of total protein lysates were separated on 10% SDS-PAGE gels and then transferred to nitrocellulose. Membranes were blocked in 5% milk/TBS/0.1% Tween-20. Primary antibodies were incubated overnight at 4°C in 5% milk/TBS, followed by a one-hour incubation of (HRP)-conjugated secondary antibody in TBS at room temperature. All antibody dilutions are listed in Table 1. HRP was inactivated by a 30 min incubation in 0.2% sodium azide in TBST at room temperature for reblotting. All blots were developed with Protoglow ECL (National Diagnostics), and densitometry analysis was performed using Fiji software (70). To quantify Akt phosphorylation, the membrane was probed with the phospho-Akt antibody and then reprobed with a 5% milk/TBS mixture including both B-actin and Akt antibodies. Per condition, phospo-Akt levels were normalized against Akt, with B-actin included as a loading control. To quantify PTEN phosphorylation, membranes were probed with phospho-PTEN or PTEN antibodies and reprobed with B-actin. Phospho-PTEN and PTEN were normalized against B-actin prior to comparison.

**Table 1:** Antibodies used in this study.

Type	Antibody Name, Source	Dilution	Source
Primary	Phospho-Akt (Thr308), Rabbit	1:2,000	#9275, Cell Signaling Technology
Primary	Akt, Rabbit	1:2,000	#9272, Cell Signaling Technology
Primary	β-actin (8H10D10), Mouse	1:4,000	#3700, Cell Signaling Technology
Primary	Phospho-PTEN (Ser380), Rabbit	1:2,000	#9551, Cell Signaling Technology
Primary	anti-PTEN (1386G), Rabbit	1:2,000	#9559, Cell Signaling Technology
Secondary	Anti-rabbit IgG, HRP-linked	1:10,000	#7074, Cell Signaling Technology
Secondary	Anti-mouse IgG, HRP-linked	1:10,000	#7076, Cell Signaling Technology
Primary	Purified Anti-PtdIns(3,4)P2 IgG, Mouse	5 ug/ml	#Z-P035, Echelon Biosciences
Primary	Purified Anti-PtdIns(4,5)P2 IgM, Mouse	10 ug/ml	#Z-P045, Echelon Biosciences
Primary	Purified Anti-PtdIns(3,4,5)P3 IgM, Mouse	20 ug/ml	#Z-P345, Echelon Biosciences
Secondary	anti-Mouse IgM (Heavy chain) Cross-Adsorbed Secondary Antibody, Alexa Alexa Fluor™ 488, Goat	1:500	#A-21042 ThermoFisher Scientific
Stain	Acti-stain 555 Phalloidin	1:50	#PHDH1, Cytoskeleton
Stain	DAPI	1:10,000	# EN62248, ThermoFisher Scientific

### 2.5 Phosphate release assay in whole cells

Cellular phosphate release was measured using a Malachite Green assay as described (39). 1 × 10^5^ neutrophils per condition were suspended in phosphate-free HBSS without calcium or magnesium (1.26 mM CaCl_2_·2H_2_O, 0.69 mM MgSO_4_·7H_2_O, 5.37 mM KCl, 136.89 mM NaCl, 5.55 mM glucose, 4.17 mM NaHCO_3_; pH 7) and were partially permeabilized for 30 s with 0.2% n-octyl-beta-D-glucopyranoside (OG) in PHEM buffer (60 mM PIPES, 25 mM HEPES, 10 mM EGTA, 2 mM MgCl2; pH 6) with Halt™ Protease Inhibitor Cocktail (Thermo Scientific). After 30 s, OG was diluted with phosphate-free HBSS (dilution factor 2), cells were centrifuged at ≥10,000 × *g* for 1 min, and the pellet was resuspended in phosphate-free HBSS (equal to starting volume). Cells were divided into microcentrifuge tubes and treated with MspA or Msp TL proteins. Free phosphate release was measured using a Malachite Green Assay Kit (#K-1500, Echelon Biosciences) according to manufacturer instructions. In short, 25 μl of phosphate standards or lysate were pipetted into the wells of a 96-well microplate in duplicate and incubated with 100 μl of Malachite Green Solution for 15 min at room temperature. Absorbance was read at 620 nm on a 96-well plate reader (Molecular Devices FlexStation3).

### 2.6 SHIP1 and PTEN immunoprecipitation and activity assays

SHIP1 and PTEN activity were determined using immunoprecipitation assays as described (33, 67). Neutrophils (5 × 10^6^ per condition) were treated with recombinant MspA or MspTL and fMLP as described, lysed with 500 μl lysis buffer (25 mM Tris pH 8.0, 150 mM NaCl, 1% Triton, 1 mM EDTA, 5% Glycerol), and immunoprecipitated using an anti-PTEN antibody (Cell Signaling, D4.3) or anti-SHIP1 antibody (Cell Signaling, D1163) overnight at 4°C, followed by binding to protein A agarose beads for 1 h (Sigma). Beads with immunoprecipitated protein were washed three times with TBS with 10 mM dithiothreitol (DTT), followed by incubation with 3,000 pmol of soluble PtdInsP_3_ (diC8-PIP3, #P3908, Echelon Biosciences) substrate for 1 h at 37°C. Samples were used in a malachite green assay to measure phosphate release as described above. Absorbance was converted into pmol phosphate using a phosphate standard curve.

### 2.7 PTEN and SHIP chemical inhibition assays

Neutrophils were isolated using phosphate-free HBSS−/− and prepared as described in “Phosphate release assay in whole cells”. Immediately before treatment with rMspA or rMspTL, cells were treated for 30 min at 37°C with or without PTEN inhibitor [2 μM SF1670 (Medchem Express)], SHIP1 inhibitor [25 μM 3α-Aminocholestane (3AC) (Millipore Sigma)], or SHIP2 inhibitor [10 μM AS1949490 (Echelon Biosciences)]. Phosphate release was then measured via Malachite Green Assay as described above.

### 2.8 Immunofluorescence

Neutrophils were isolated and treated with 100 nM of recombinant protein (2 × 10^6^ PMNs per condition in 200 μl HBSS). 100 μl of cells were moved to sterile coverslips (2 coverslips with 1 × 10^6^ PMNs each per condition) and left for 30 min at room temperature to allow for attachment. The supernatant was removed, and cells on coverslips were incubated with 200 μl of 1 uM fMLP at room temperature for 5 min; then fixed with 300 μl 4% paraformaldehyde for 20 min. Coverslips were washed three times with 400 μl TBS, and cells permeabilized with 300 μl 0.5% Saponin (Thermo Scientific Chemicals) for 15 min at room temperature. Coverslips were washed thrice with TBS and then blocked with 400 μl of 10% Normal Goat Serum (NGS) in TBS either overnight at 4°C or 30 min at 37°C. Cells were then stained with an individual anti-PIP antibody diluted in 10% NGS at concentrations indicated in Table 1 for 60 min at 37°C. Following incubation, coverslips were washed for 5 min with 300 ul TBS-NGS 1%, 3 times, and then stained with secondary antibodies and stains, as listed in Table 1, in 10% NGS in TBS in the dark for 45 min at room temperature**.** Coverslips were washed thrice for 5 min with 300 ul TBS-NGS 1%, then mounted with DAKO mounting media to a microscope slide. Slides were stored in the dark at 4°C until imaging. Cells were imaged using an Andor Dragonfly spinning disk confocal microscope, with five images taken per coverslip. The mean gray value (average pixel intensity in a given area, in this case a cell) was calculated for each whole cell to determine the florescence of each cell. This analysis was performed using Fiji macros; a cell was defined as an object with an area of at least 10 pixles^2^ and a roundness of at least 0.3 (71). Figures were prepared with the QuickFigures ImageJ plugin (72).

### 2.9 Quantification of Pi(4,5)P_2_ from neutrophils and PIP5K inhibition study

Neutrophils (5 × 10^6^ cells per condition) were exposed to recombinant 100 nM rMspA or MspTL for 30 min. In some experiments, neutrophils were pre-treated with 30 μM ISA-2011B (#50-202-9220, ThermoFisher Scientific) for 1 h at 37°C to inhibit PIP5K activity, prior to treatment with rMspA or rMspTL. Phosphoinositides were isolated using the NeoBeads PIP Purification System (#P-B999, Echelon Biosciences), following the manufacturer's instructions. 20 mg beads were reconstituted in 150 μl dH_2_O, and 1 mg of beads (7.5 μl slurry) was added to each condition. Extractions were performed in disposable borosilicate glass culture tubes. PI(4,5)P_2_ was quantified using the PI(4,5)P_2_ Mass **e**nzyme-**l**inked **i**mmuno**s**orbent **a**ssay (ELISA) Kit (#K4500, Echelon Biosciences) according to the manufacturer's instructions. All samples and standards were run in duplicate. Plates were read at 450 nm on a 96-well plate reader (Molecular Devices FlexStation3). PI(4,5)P_2_ (diC16) was used to construct standard curves using non-linear regression analysis with GraphPad Prism software, and data was analyzed with a sigmoidal dose-response with variable slope curve analysis (four-parameter, 4PL curve fit).

### 2.10 Small GTPase-protein activation assays

The activity of small GTPases (Rac1, RhoA, Cdc42) in response to treatment with recombinant Msp, MspA, and MspTL were measured with commercially available G-LISA assays following the manufacturer's instructions (Rac1 #BK126, RhoA #BK121, Cdc42 #BK127, Cytoskeleton Inc.). 5 × 10^6^ neutrophils per condition were treated with recombinant protein prior to fMLP exposure for 1 min. Cells were lysed in 140 μl of the provided lysis buffer following the manufacturer's instructions. 100 μl of lysate was snap-frozen in liquid nitrogen and stored at −80°C until further use, and 40 μl of lysate was used immediately to measure protein concentrations as described in the assay protocol. Cell lysates containing equal amounts of protein were used between conditions of all assays. Assay endpoints were measured using a 96-well plate reader (Molecular Devices FlexStation3).

### 2.11 Statistical analysis

Comparisons between two groups were performed by paired or unpaired *t*-tests, as appropriate. Following normality tests (Shapiro–Wilk, significance 0.05), comparisons between more than two groups were performed by ANOVA with *post-hoc* Tukey HSD or Dunn's multicomparison tests, as appropriate. For immunofluorescence data, outliers were identified by the ROUT method, Q = 0.5%. All statistical analyses were performed using GraphPad Prism software (GraphPad, San Diego, CA). Results are based on at least 3 independent experiments, as indicated by the *n*-value in each figure legend, and are shown as individual points on graphs. Statistical significance was defined as a *p* value of <0.05. Error bars represent the standard errors of the means (SEM).

## 3 Results

### 3.1 MspA and MspTL disorder phosphoinositide production in neutrophils

**Figure 1:**
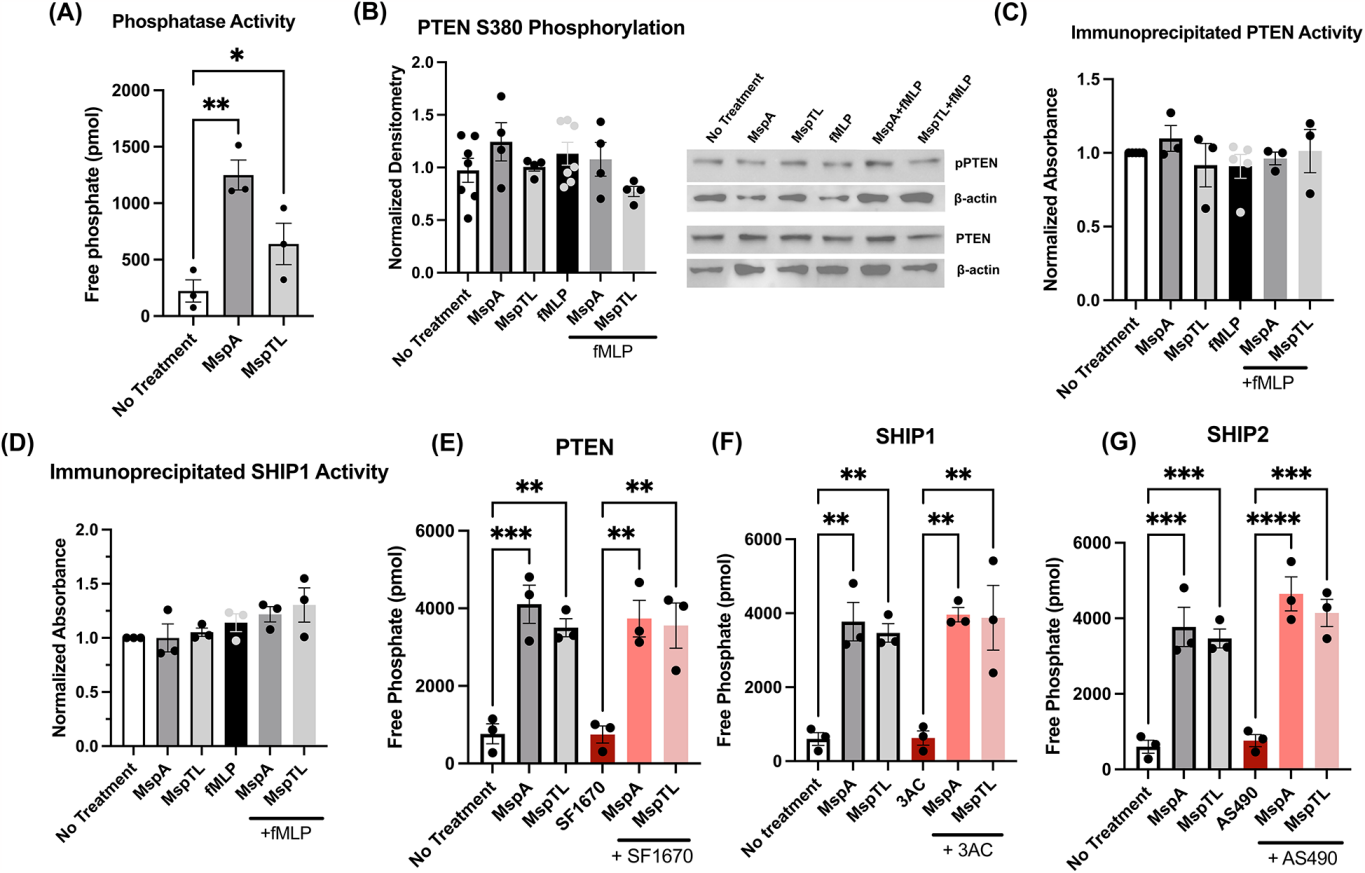
MspA and MspTL increase phosphatase activity, but not via PTEN or SHIP activity. **(A–D)** Mouse bone marrow neutrophils were isolated, treated with 100 nM of recombinant MspA or MspTL for 30 min, then stimulated with 1 µM fMLP for 1 min prior to assays. **(A)** Total phosphatase activity was measured in neutrophil whole cell lysates by malachite green assay. **(B)** PTEN phosphorylation (Ser380) was assessed by immunoblot as a measure of activation. Neutrophils untreated or stimulated with fMLP served as negative and positive (+fMLP) controls while β-actin was included as a loading control. Right shows the mean ± SEM of 6 independent experiments, left shows a representative blot. **(C)** PTEN and **(D)** SHIP1 were immunoprecipitated from neutrophil lysate and then used in malachite green assays. **(E–G)** Mouse bone marrow neutrophils were isolated and treated with **(E)** 2 μM SF1670 (PTEN inhibitor), **(F)** 25 μM 3AC (SHIP1 inhibitor), or **(G)** 10 μM AS1949490 (SHIP2 inhibitor) for 30 min at 37C, then treated with 100 nM of recombinant MspA or MspTL for 30 min. Total phosphatase activity was measured in whole cell lysate by malachite green assay. Graphs show mean ± SEM of at least 3 independent experiments, with dots representing biological replicates. *p < 0.05, **p < 0.01, ***p < 0.001, ****p < 0.0001 by ANOVA, **(A)** *F*_5, 12_ = 8.969; **(B–D)** n.s.; **(E)** *F*_5, 12_ = 14.08, **(F)** *F*_5, 12_ = 13.39, **(G)** *F*_5, 12_ = 25.90.

Intracellular phosphoinositide levels are tightly regulated by lipid kinases and phosphatases. *T. denticola* Msp has been shown to disrupt this balance by increasing activity of the phosphatase PTEN, which dephosphorylates PIP_3_ to form PI(4,5)P_2_, and inhibiting activity of PI3-Kinase, which phosphorylates PI(4,5)P_2_ to form PIP_3_ (67). Thus, we first assessed total phosphatase activity in partially permeabilized neutrophils using a malachite green assay and found that both MspA and MspTL significantly increased free phosphate release in neutrophils (Figure 1A). Knowing that Msp impacts PTEN regulation by altering phosphorylation (33, 67), we examined PTEN phosphorylation at Serine 380 by immunoblotting. Surprisingly, unlike Msp, neither MspA nor MspTL significantly affected PTEN phosphorylation at S380 (Figure 1B). Using specific PTEN (Figure 1C) and SHIP1 (Figure 1D) immunoprecipitation assays, PTEN and SHIP1 were immunoprecipitated from MspA- and MspTL-treated neutrophils, and the amount of PIP_3_ converted to PIP_2_ was measured using a malachite green phosphatase assay. However, there was no significant difference in PIP_2_ production by either PTEN or SHIP1. To verify this result, partially permeabilized neutrophils were pretreated with specific chemical inhibitors for PTEN (SF1670; Figure 1E), SHIP1 (3AC, Figure 1F), or SHIP2 (AS490, Figure 1G) before exposure to Msp proteins and phosphate release was again measured via malachite green assay. Inhibition of either of these three phosphatases did not prevent MspA or MspTL from increasing free phosphate levels. Overall, this data implies that MspA- or MspTL-mediated increase in free phosphate is not due to modulation of PTEN or SHIP activity.

**Figure 2:**
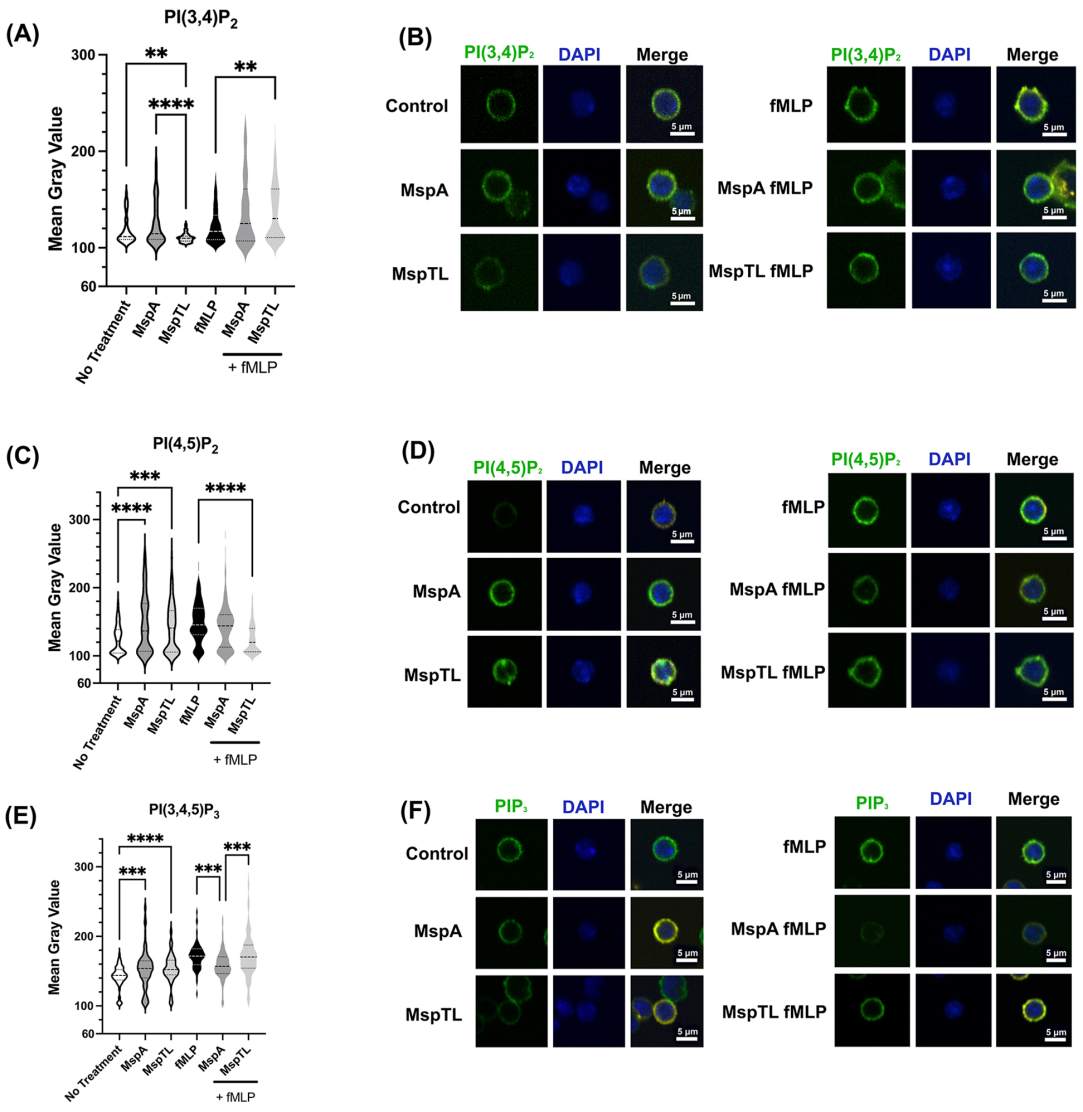
MspA and MspTL proteins alter phosphoinositide levels. Murine bone marrow neutrophils were isolated, exposed to 100 nM of rMspA or rMspTL for 30 min or exposed to proteins then stimulated with 1 μM fMLP for 1 min. Cells were fixed to coverslips and stained for DAPI (blue) or individual phosphoinositide isoforms (green). Representative images showing antibody staining for **(A)** PI(3,4)P2 **(B)** PI(4,5)P2 or **(C)** PIP3. Graphs show average mean gray value (MGV) of 3 independent experiments next to representative images. MGV was calculated for each whole cell. Dotted lines indicate quartiles and dashed lines indicate medians. **p < 0.01, ***p < 0.001, ****p < 0.0001 by ANOVA, **(A)** H(5) = 86.58, **(B)** H(5) = 76.79, **(C)** H(5) = 161.1.

Next, we examined the effects of MspA and MspTL on individual phosphoinositide levels in neutrophils. Initially, we used immunofluorescence analysis with antibodies against specific PIP species as a relative quantitative measure. MspTL exposure significantly decreased PI(3,4)P_2_ fluorescence in comparison to both untreated cells and MspA-treated cells (Figures 2A,B), though both MspA and MspTL increased PI(4,5)P_2_ (Figures 2C,D) and PIP_3_ fluorescence (Figures 2E,F). In response to fMLP stimulation, MspTL pre-treatment enhanced PI(3,4)P_2_ production (Figures 2A,B), yet inhibited PI(4,5)P_2_ production (Figures 2C,D). In contrast, both MspA and MspTL pre-treatment inhibited PIP_3_ production (Figures 2E,F) downstream of fMLP stimulation.

### 3.2 MspA and MspTL increase PI(4,5)P_2_ *via* PIP5K

**Figure 3:**
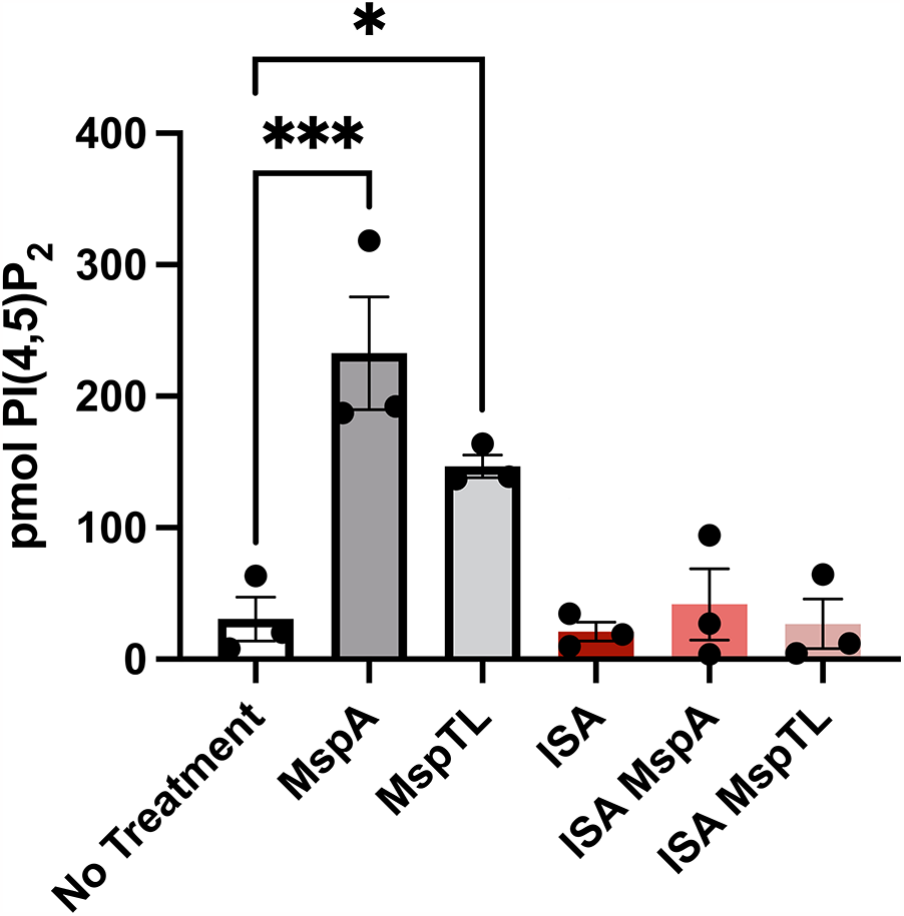
Inhibiting PIP5K prevents MspA and MspTL from increasing PI(4,5)P2 levels. Murine bone marrow neutrophils were treated with or without 30 μM of the PIP5K inhibitor ISA-2011B for 1 h at 37C followed by exposure to 100 nM of rMspA or rMspTL for 30 min. PIPs were isolated using NeoBeads PIP purification system and quantified with PI(4,5)P2 Mass ELISA. Graph shows mean ± SEM of 3 independent experiments, each point represents a biological replicate. *p < 0.05, ***p < 0.001 by ANOVA, F_5, 12_ = 13.62.

Considering that MspA and MspTL treatment increased PI(4,5)P_2_ intensity by microscopy (Figure 2), PI(4,5)P_2_ is found at a higher density than other phosphoinositide species in the PM (55), and that it is affected by *T. denticola* Msp (67), we wanted to more accurately quantify PI(4,5)P_2_ levels. Using PIP purification NeoBeads together with a PI(4,5)P_2_ Mass ELISA, we noted a significant increase in PI(4,5)P_2_ levels in neutrophils treated with MspA (7.6 fold) or MspTL (4.8 fold) compared to untreated neutrophils (Figure 3) (55). As our data suggests that MspA and MspTL do not increase PTEN activity (Figure 1), we assessed additional pathways of PI(4,5)P_2_ metabolism. PIP5K can synthesize PI(4,5)P_2_ through PI(4)P phosphorylation. Thus, we examined PI(4,5)P_2_ production in the presence or absence of the PIP5K chemical inhibitor ISA-2011B (73). There are three isoforms of PIP5K in mammals: PIP5Kα, PIP5Kβ, and PIP5Kγ. Mouse (74) and human (75) PIP5K isozymes were cloned by independent laboratories in parallel resulting in differing nomenclature: human PIP5Kα and PIP5Kβ, respectively, correspond to mouse PIP5Kβ and PIP5Kα. As such, ISA-2011B inhibits PIP5Kα in humans and PIP5Kβ in mice. Pretreatment with ISA-2011B completely prevented MspA and MspTL from increasing PI(4,5)P_2_ levels (Figure 3), suggesting the MspA and MspTL-mediated increase in neutrophil PI(4,5)P_2_ is PIP5K dependent.

### 3.3 MspA and MspTL inhibit neutrophil intracellular signaling downstream of PIP_3_

**Figure 4:**
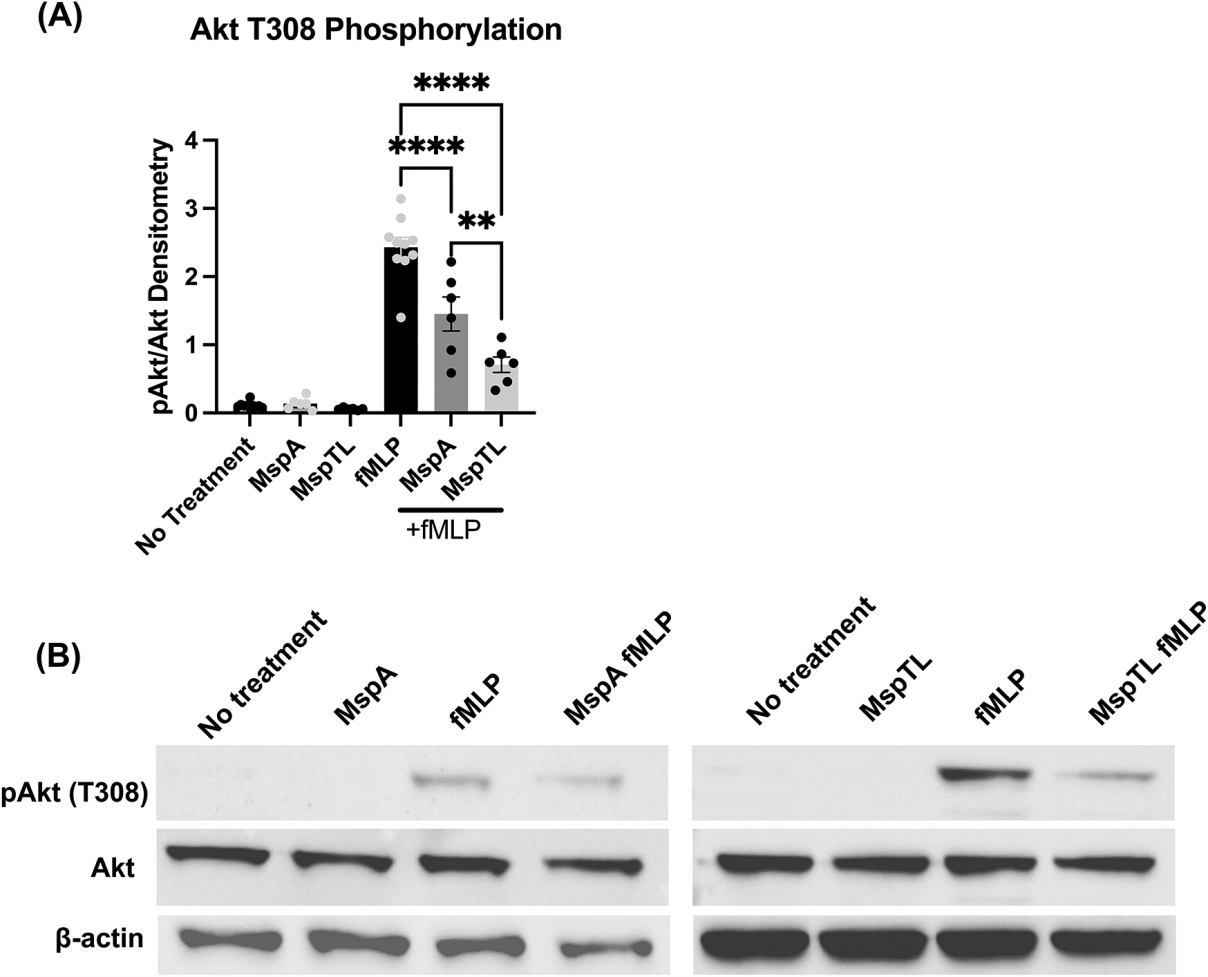
MspA and MspTL inhibit Akt phosphorylation. Murine bone marrow neutrophils were treated with 100 nM purified recombinant MspA or MspTL for 30 min at RT then stimulated with 1 μm fMLP for 1 min. Akt phosphorylation at Thr308 was assessed by immunoblot as a measure of activation. Neutrophils untreated or stimulated with fMLP served as negative and positive (+fMLP) controls, with β-actin included as an additional loading control. **(A)** Densitometry graph of Akt phosphorylation at T308 with a **(B)** representative immunoblot. **p < 0.01, ****p < 0.0001 by one-way ANOVA, *F*_5, 38_ = 71.53.

Appropriate recruitment of PIP_3_ binding effectors at the plasma membrane pathway regulates numerous neutrophil functions, including chemotactic directionality (76, 77). Downstream of PI3K activation, Akt is allosterically activated by the binding of PIP_3_ to its PH domain leading to phosphorylation (78). Akt phosphorylation is considered an indirect measure of PIP_3_ generation, thus Threonine 308 phosphorylation was assessed by immunoblotting. While fMLP stimulation increased Akt phosphorylation as expected, neither MspA nor MspTL exposure alone induced significant Akt phosphorylation. However, in line with our immunofluorescence data (Figures 2E,F) both MspA and MspTL significantly prevented fMLP-induced Akt phosphorylation, with MspTL having a greater inhibitory effect than MspA (Figure 4).

**Figure 5:**
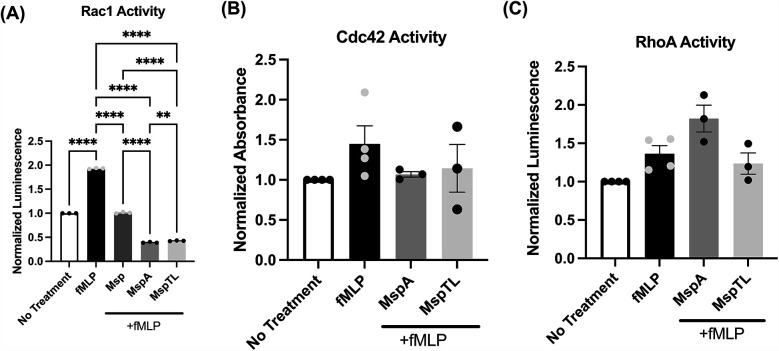
MspA and MspTL inhibit Rac1 activity. Total activity of **(A)** Rac1 **(B)** Cdc42 and **(C)** RhoA were measured in neutrophils treated with or without 100 nm recombination Msp proteins for 30 min. and with or without 1 um fMLP for 1 min by G-LISA. All graphs show mean ± SEM of 3 independent experiments, with each point representing a biological replicate. **p < 0.01, ****p < 0.0001 by ANOVA, **(A)** F_4, 10_ = 12,791, **(B, C)** n.s.

The small Rho family of GTPases are molecular switches controlling essential cellular functions and processes including migration and actin remodeling (79). Rac1 is crucial for lamellipodia formation, requiring PIP_3_ for activation (80), and is selectively inhibited by Msp (66). Small G-protein Activation Assays (G-LISAs) were used to measure the activity of the Rho family GTPases Rac1, RhoA, and Cdc42. Both MspA and MspTL significantly prevented Rac1 activation in response to fMLP, to an even greater extent than Msp (Figure 5A). MspTL did not significantly prevent Cdc42 (Figure 5B) or RhoA (Figure 5C) activation. There was a minor yet non-significant trend of MspA to impair Cdc42 activation downstream of fMLP stimulation (Figure 5B) while RhoA was not affected (Figure 5C).

### 3.4 MspA and MspTL modify actin localization in neutrophils

**Figure 6:**
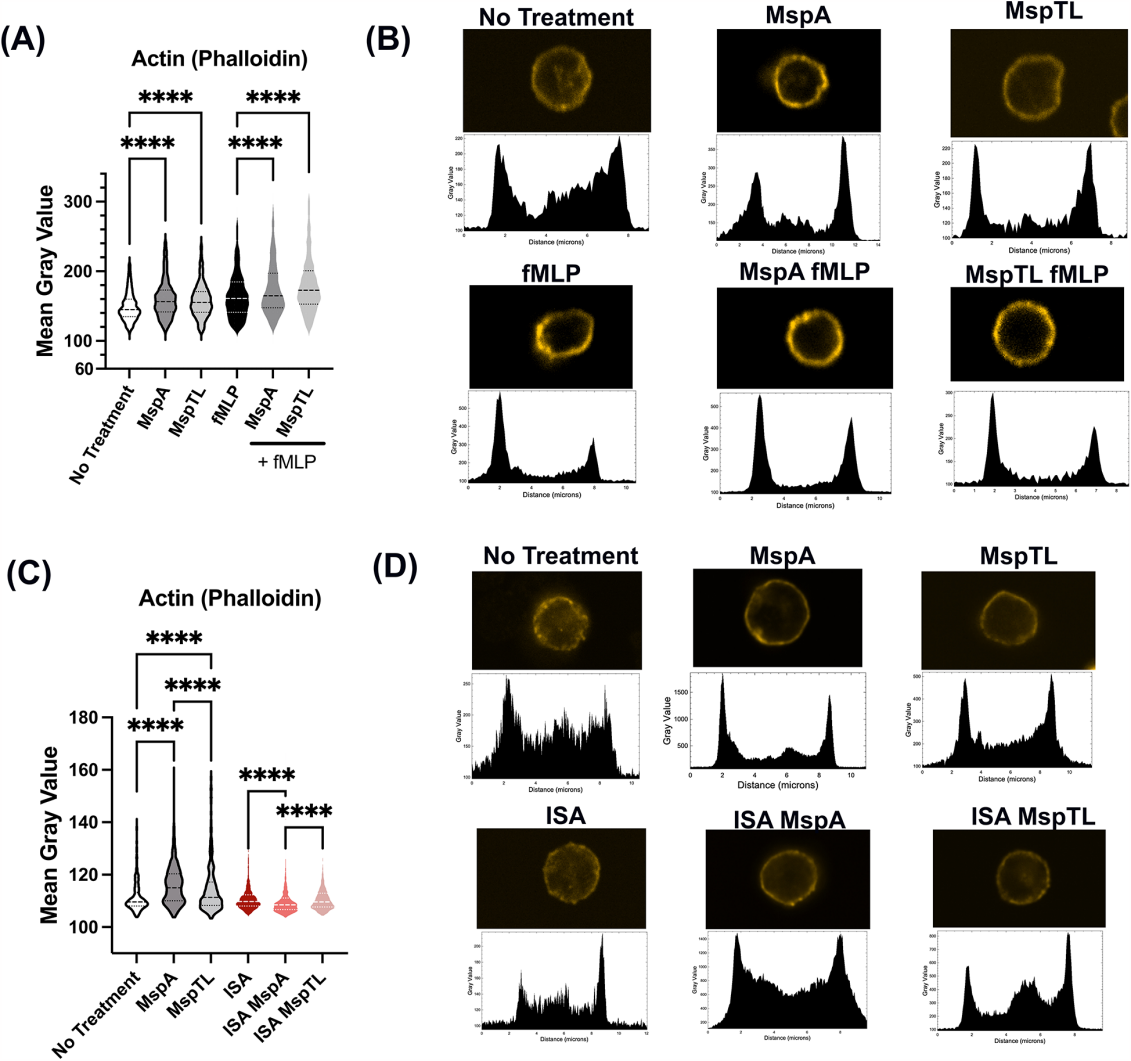
MspA and MspTL increase cortical actin in neutrophils via PIP5K. Murine bone marrow neutrophils were isolated, **(A,B)** treated with 100 nM of MspA or MspTL for 30 min, then stimulated with 1 μM fMLP for 1 min at RT or **(C,D)** Treated with 30 μM ISA-2011B for 1 h at 37C followed by 100 nM MspA or MspTL. Cells were fixed to coverslips and stained for f-actin. Shows **(A,C)** mean gray values (MGV) of **(A)** 6 or **(C)** 3 independent experiments, dotted lines indicate quartiles and dashed lines indicate medians. MGV was calculated for each cell. **(B,D)** show representative single cell images (top) and their corresponding profile plot (bottom). ****p < 0.0001 by ANOVA, **(A)** H(5) = 470.5, **(C)** H(5) = 615.1.

Appropriate PIP recruitment and cell signaling drive the dynamic actin rearrangements that underlie neutrophil functionality (81, 82). F-actin polymerization and localization were assessed by immunofluorescent microscopy of phalloidin in MspA and MspTL-treated neutrophils. Exposure to either MspA or MspTL alone or pretreatment of fMLP-stimulated neutrophils with MspA or MspTL significantly enhanced the intensity of F-actin as measured by immunofluorescence (Figure 6A). Plot profile analysis reveals that actin is localized predominately at the cell periphery (Figure 6B), indicating a thickening of the actin cell cortex. PI(4,5)P_2_ regulates actin filament rearrangement by sequestering free G-actin monomers, promoting F-actin assembly, and binding numerous actin regulatory proteins (83). Since our data indicates MspA and MspTL increase PI(4,5)P_2_ levels (Figure 3) in a PIP5K-dependent manner, we next assessed F-actin phalloidin staining following exposure to the PIP5K inhibitor ISA-2011b. In these experiments, MspA or MspTL alone increased F-actin intensity and cortical distribution yet pre-treatment with ISA-2011b prevented MspA or MspTL-mediated increase in F-actin levels (Figures 6C,D).

## 4 Discussion

Periodontitis is driven by complex interactions between bacteria and periodontal tissues, including immune cells, where bacterial surface proteins directly interact with host cells. Numerous *Treponema* species are prevalent in the subgingival microbial community during severe periodontitis and remain present both in individuals with persistent aggressive disease and at non-responsive treatment sites (84, 85). The impact of *T. denticola* and its virulence factor Msp on multiple host cell types has been well-documented (29, 33, 38, 39, 41, 66, 86–89). Despite their persistence during periodontitis, *T. maltophilum* and *T. lecithinolyticum* virulence factors remain poorly understood. Previous research has found that *T. maltophilum* MspA and *T. lecithinolyticum* MspTL proteins induce the release of pro-inflammatory cytokines from monocytes and fibroblasts (44, 47), while MspTL also increases ICAM1 expression and monocyte adhesion to microvascular endothelial cells (47). We have recently shown that MspA and MspTL inhibit neutrophil chemotaxis (46). In this study, we characterize the molecular effects of the *Treponema* outer membrane proteins MspA and MspTL on neutrophil PIP-associated intracellular signaling.

Appropriate neutrophil responses are required to maintain periodontal health and are in part driven by complex regulatory signaling pathways regulated by phosphoinositide metabolism (55, 90–92). Our previous work, demonstrating that Msp disorders activity of the PI3K/PTEN signaling axis and downstream mediators to impair chemotaxis, led us to target this pathway (33, 40, 67). Interestingly, our results herein indicate that despite increasing cellular phosphate release neither MspA nor MspTL appear to modulate activity of the phosphoinositide phosphatases PTEN or SHIP. Our imaging analysis revealed that both MspA and MspTL modulate the intensity of distinct PIP species at the plasma membrane, including PI(4,5)P_2_, supported by our quantitative measurements. However, there are limitations in the interpretation of our current quantification data. While the visualization of transfected PIP-binding domains as molecular biosensor probes for cellular lipid localization are often used, they are not without weaknesses. Antibody-mediated PIP staining remains a valid measure for estimation of subcellular localization and relative quantification particularly when well-characterized antibodies are used with appropriate fixation methods to retain plasma membrane and lipid integrity (93, 94). Quantification of cellular PIPs by techniques such as mass spectrometry has advanced, yet remains technically specialized, and there are limitations in comprehensively measuring all variants and detecting small changes in low abundance species (95, 96). Thus, biochemical approaches such as enrichment of total PIPs from neutrophils using neomycin beads [NeoBeads (97)] together with a specific PIP detector protein in a competitive ELISA-based format (as utilized here), provide robust data. Overall, our microscopic observations together with quantitative biochemical analysis give confidence that PI(4,5)P_2_ production is increased by these *Treponema* proteins. However, further study is needed to more accurately quantify and define temporal and spatial dynamics of PIPs including PI(3,4)P_2_ and PIP_3_ following MspA and MspTL exposure.

PI(4,5)P_2_ is the most abundant plasma membrane phosphatidylinositol and a crucial intracellular signaling molecule. It serves as a precursor for phospholipase C (PLC)–generated and PI3K–generated messengers, directly regulates the activity of many integral membrane ion channels and transporters, acts as an anchor point for other proteins at the membrane, and contributes to membrane and cytoskeleton remodeling (55). While PI(4,5)P_2_ can be produced from PIP_3_ through PTEN activation, PI(4,5)P_2_ is predominately synthesized by PI4P 5-kinases (PIP5K) from phosphatidylinositol 4-phosphate (PI4P) (98) or in lesser amounts by PI5P 4-kinases (PIP4K) from phosphatidylinositol 5-phosphate (PI5P) (99). There are three isoforms of PIP5K in mammals: PIP5Kα, PIP5Kβ, and PIP5Kγ, each with many splice variants (98). Conflicting PIP5K nomenclature exists, where mouse PIP5Kβ is equivalent to human PIP5Kα and vice versa, and as this study used murine neutrophils, we use the isoform nomenclature for the mouse protein (74, 75, 100). PIPI5K isoforms demonstrate unique subcellular localization in different cell and tissue types, mediating temporal pools of PIP_2_ to orchestrate distinct cellular functions. For example, mouse PIP5Kα (human PIP5Kβ) has been observed at nuclear vesicles (101), mouse PIP5Kβ (human PIP5Kα) localizes primarily to the plasma membrane (102), and PIP5Kγ can localize to intracellular membrane compartments, focal adhesion complexes, and intracellular connections (103). Our experiments using the chemical PIPK5K inhibitor ISA-2011B (which inhibits mouse PIP5Kβ) (73) to prevent MspA and MspTL-mediated PI(4,5)P_2_ production indicates that the PIP5Kβ isoform is required for the activity of these proteins in neutrophils. Additional research is needed to further delineate how MspA and MspTL affect individual PIP5K isoforms, if they directly manipulate PI(4,5)P2-associated protein activity or transcription, or if the observed effects are solely due to altered phosphoinositide production or other changes at the plasma membrane. While *T. denticola* Msp does not affect the transcription of PIP phosphate kinases we have examined to date (33), our work herein indicates that MspA and MspTL differ from Msp in their upstream effect on cellular pathways, therefore we cannot exclude effects on transcription. Differences between Msp-like protein functionality could occur, as we have noted differences in their protein topological features (46).

Phosphoinositide-associated signaling regulates the dynamic actin cytoskeletal remodeling that is required for neutrophil functions including chemotaxis. Bacterial pathogens can disrupt phosphoinositide signaling and actin dynamics at the host's plasma membrane to promote cell infection and modulate function (104–108). *T. denticola* Msp modulates actin remodeling dynamics in a PIP_2_-dependent manner (33, 67), and we extend these findings by demonstrating that MspA and MspTL increase the overall intensity of cortical F-actin in neutrophils. Furthermore, our chemical inhibition studies reveal that MspA and MspTL-mediated F-actin changes are PIP5K-dependent. In neutrophil-like HL60 cells, human PIP5Kα (mouse PIP5Kβ) and γ accumulate at the leading edge (109) while human PIP5Kβ (mouse PIP5Kα) localizes to the uropod and interacts with actin-membrane linking ERMs (ezrin, moesin, radixin) proteins, which in turn inhibit RhoGDI and lead to RhoA activation (110–112). PIP5Kγ also localizes to the uropod in mouse neutrophils where its kinase activity is necessary for chemotaxis (113) and the PIP5KIγ90 variant is polarized by interaction with neutrophil integrins (114). Both PIP5Kα (human PIP5Kβ) and γ isoforms are recruited to the neutrophil uropod (trailing end) during polarization to contribute to cell retraction yet have distinct functions within this process (110, 113). While much focus is on impairment of the leading-edge formation (“frontness”) required for neutrophil polarization as a prerequisite to chemotaxis; including following exposure to Msp through disruption of PIP_3_ accumulation and Rac1 activation (40, 66, 67), improper molecular recruitment and signaling at the uropod (“backness”) through disrupting PIP5K interactions could impair migration and neutrophil function. Local synthesis of PI(4,5)P_2_ by the PIP5KIγ90 isoform supports *S. aureus* invasion of human cells (115) and this same PI5K isoform is involved in integrin-induced neutrophil polarization and migration *in vivo* (114). To our knowledge, no reports define a role for bacterial-mediated manipulation of PIP5Kβ signaling, so this may represent a novel molecular process by these proteins.

Effective signaling and regulatory pathways downstream of receptor engagement through PIP-binding effectors are crucial for cellular actin-mediated functions. Rho family small GTPases are master regulators of the actin cytoskeleton and neutrophil function with PIP3 mediating their recruitment and function at the plasma membrane (79). Like *T denticola* Msp, MspA and MspTL prevent Rac1 activation while allowing activation of RhoA and Cdc42 downstream of fMLP stimulation, showing the neutrophils can still detect the chemoattractant. This suggests Msp-like proteins from different *Treponema* species selectively modify distinct regulators of Rac1 and/or localization at the plasma membrane, yet how this happens molecularly remains unsolved. Polarized localization of Rac1 requires membrane translocation dependent on specific plasma membrane phospholipid nanocluster spatial localization and charge-mediated electrostatic interactions preceding nucleotide exchange (116, 117). Activation of small GTPases requires nucleotide exchange factors (GEF), of which Rac1-specific GEFs have been identified.

In this work, we show via phalloidin immunostaining that MspA and MspTL increase the overall intensity of neutrophil F-actin at the PM dependent on PIP5K activity. Many proteins responsible for actin cytoskeleton remodeling at the cell cortex are regulated by PI(4,5)P_2_-binding effector molecules (118). Msp modifies actin filament formation and uncapping through release of gelsolin and CapZ protein in neutrophils (67).

While we have not defined specific mechanisms for actin reorganization in this work, future studies will elucidate MspA/MspTL-mediated actin-associated pathways regulated by the PIP5K-PI (4,5)P_2_ axis in neutrophils. For example, in addition to filament uncapping proteins, actin branching networks such as the WASP/ARP2/3 complex important for actin remodeling at the leading edge could be impacted (119). Actin-binding proteins such as ezrin are required for cortical actin cytoskeleton and plasma membrane cross-linked organization (120). In neutrophils, ERM proteins are regulated by PIP5K and RhoA interaction at the uropod to control cell retraction and interaction with the substratum (110), thus disruptions to these signaling networks could cause changes in physiological cortical actin remodeling.

A question remaining is what the source(s) of the observed increased free phosphate from neutrophils is. The malachite green assay we utilized is based on the principle of formation of a complex in the presence of malachite green, molybdate, and free orthophosphate (121). This assay is commonly used to measure phospholipid phosphatase activity (122) and we presumed our results would demonstrate PTEN/SHP activity releasing free inorganic phosphate during PI (4,5)P_2_ production. However, in principle the free phosphate detected could represent activity of other cellular lipid phosphatases, protein phosphatases, enzymes or nucleoside triphosphatases. Protein phosphorylation is one of the most common cellular post-translational modifications regulating cellular processes (123). It is possible that MspA/MspTL could affect the phosphorylation state of numerous signaling pathways to impact neutrophil behavior and this is an area to examine. In terms of PIP5K, Msp proteins may modify phosphorylation events to change kinase activity or protein interactions. For example, phosphorylation of PIP5Kβ at Ser214 reduces its activity (124) while PIP5KIγ phosphorylation regulates its polarization and interaction with FAK proteins (125). PIP5K can also autophosphorylate its Ser/Thr residues, inhibiting its own activity *in vitro* (126). We hope to, in our future studies, identify the unknown source behind the observed increases in free phosphate and eludicate the effects of MspA and MspTL on PIP5K isoforms.

We initially hypothesized that MspA and MspTL disrupt phosphoinositide signaling through modulation of PTEN activity to dysregulate downstream neutrophil function. However, we show that MspA and MspTL increase phosphate release in neutrophils, but that unlike Msp, this is not a result of PTEN (or SHIP) activity. Instead, while MspA and MspTL differently modulate phosphoinositide species, both increase PI (4,5)P_2_ in a PIP5K-dependent manner, inhibit Akt phosphorylation, decrease Rac1 activity, and modify cortical actin distribution. Awareness of the mechanisms by which these understudied bacteria manipulate neutrophil signaling and function are crucial for the development of therapies to halt the progress of periodontal disease and improve oral health outcomes.

## Data Availability

The raw data supporting the conclusions of this article will be made available by the authors, without undue reservation.
