# Supplementary figures and images for: Emerging oral Treponema membrane proteins disorder neutrophil phosphoinositide signaling via phosphatidylinositol-4-phosphate 5-kinase
Source: Front Oral Health. 2025 Apr 3;6:1568983. doi: 10.3389/froh.2025.1568983 (PMC12003349; doi:10.3389/froh.2025.1568983)

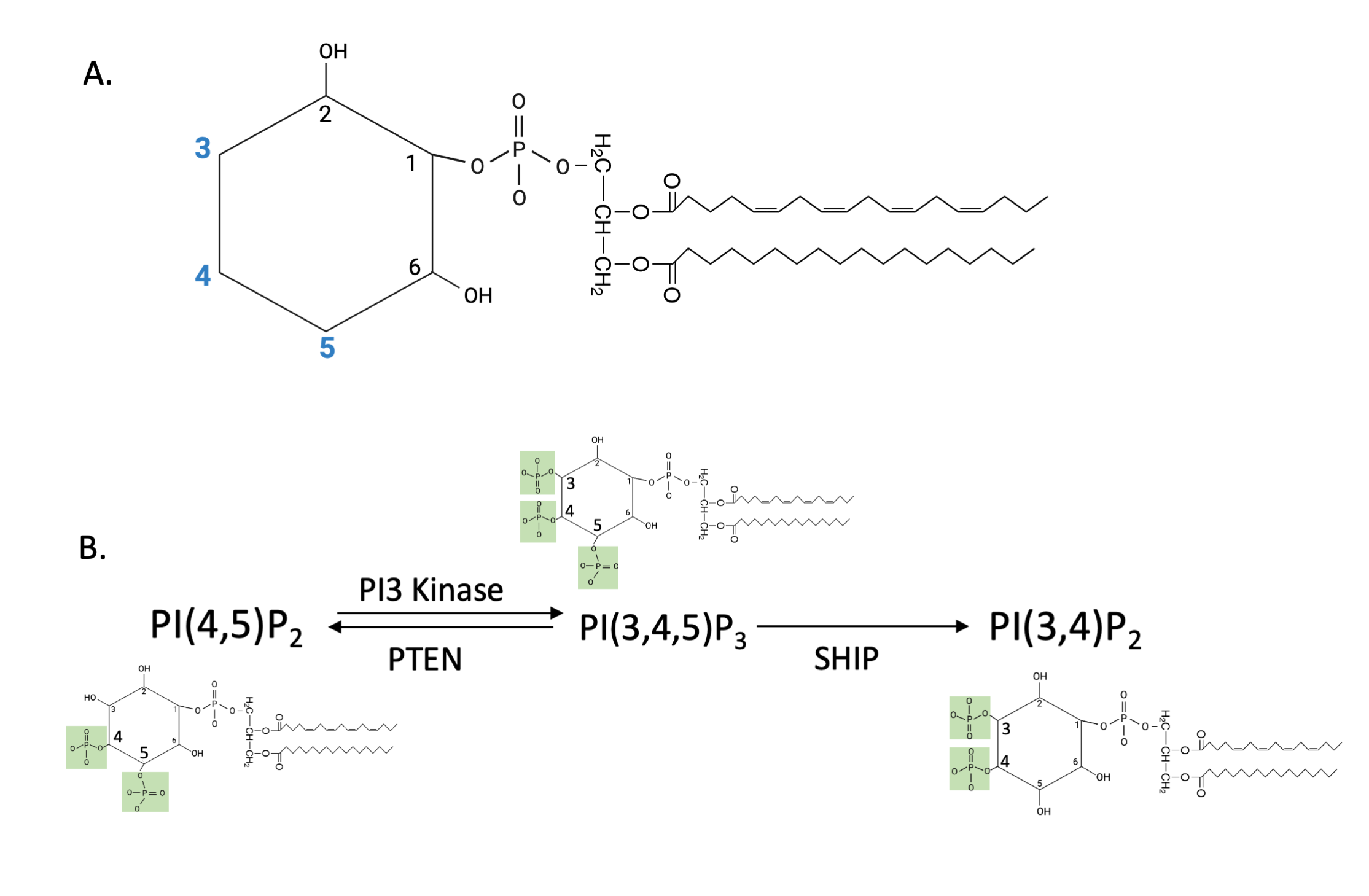

Supplement: Supplementary file 1 [file Image1.tiff]

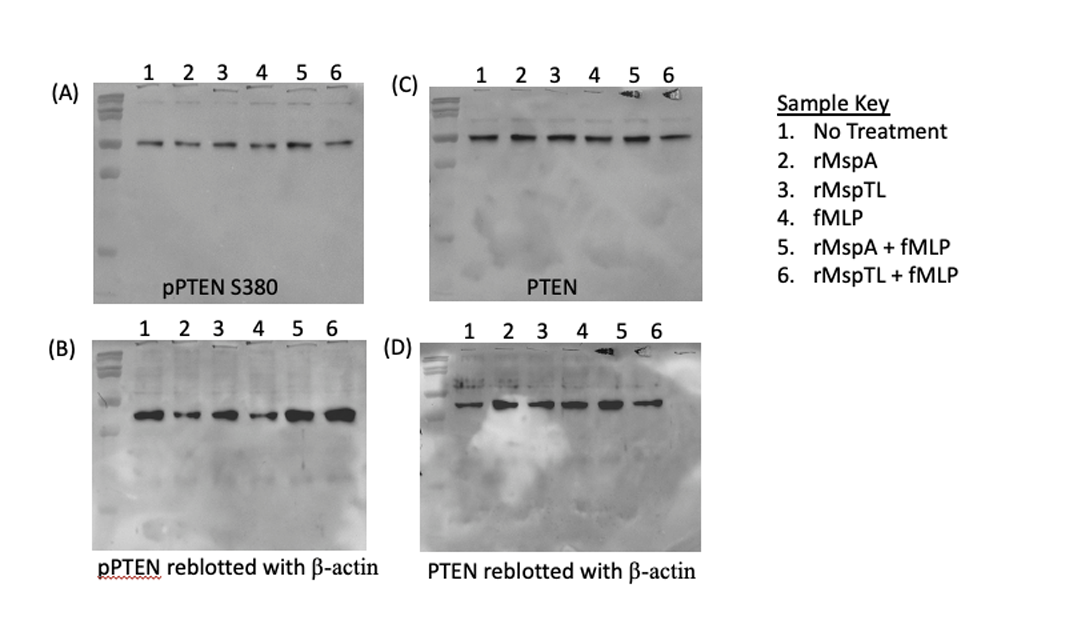

Supplement: Supplementary file 2 [file Image2.png]

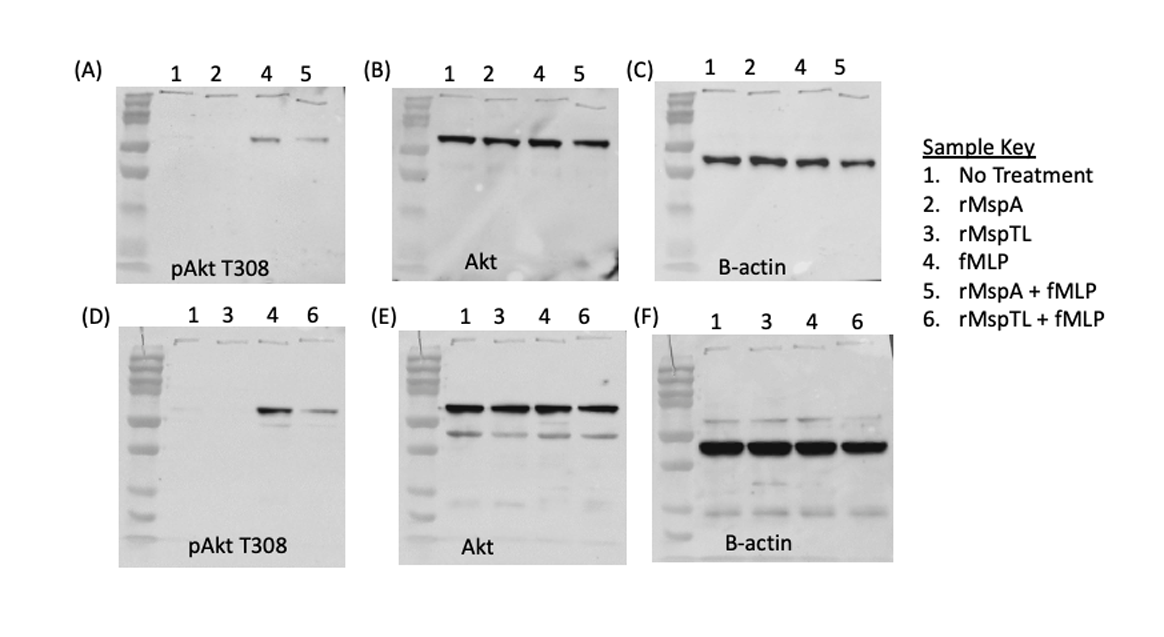

Supplement: Supplementary file 4 [file Image4.png]
