# Supplementary material for: Emerging oral Treponema membrane proteins disorder neutrophil phosphoinositide signaling via phosphatidylinositol-4-phosphate 5-kinase
Source: Front Oral Health. 2025 Apr 3;6:1568983. doi: 10.3389/froh.2025.1568983 (PMC12003349; doi:10.3389/froh.2025.1568983)
Supplement: Supplementary file 6 [file Table1.docx]

**Supplementary Figure 1: Phosphoinositide structure and PIP_3_ dynamics.** (A) A generic phosphatidylinositol molecule, where the head group can contain either a phosphate or hydroxyl group at positions 3, 4, and 5 (blue) to form different phosphoinositide species. (B) Schematic of PIP_3_ dynamics, showing the structures of each PIP molecule with the phosphate groups highlighted in green.

**Supplementary Figure 2: Uncropped images of western blots presented in Figure 1B.** Murine neutrophils were treated with 100 nM of recombinant MspA or MspTL for 30 min, then stimulated with 1 μM fMLP for 1 minute where noted. Neutrophils untreated or stimulated with fMLP served as negative and positive (+fMLP) controls. Lysates were blotted against A) pPTEN Ser380 or B) total PTEN antibody on separate membranes. HRP was inactivated followed by reblotting of each membrane with a (C,D) β-actin antibody as a loading control.

**Supplementary Figure 3: Enlarged images of PIP florescent microscopy presented in Figure 2.** Murine neutrophils were treated with 100 nM of recombinant MspA or MspTL for 30 min, then stimulated with 1 μM fMLP for 1 minute where noted. Neutrophils untreated or stimulated with fMLP served as negative and positive (+fMLP) controls. Cells were fixed to coverslips and stained for a phosphoinositide species ( PI(3,4)P_2_, PI(4,5)P_2_, or PIP_3_), shown in green.

**Supplementary Figure 4: Uncropped images of western blots presented in Figure 4.** Murine neutrophils were treated with 100 nM of recombinant MspA or MspTL for 30 min, then stimulated with 1 μM fMLP for 1 minute where noted. Neutrophils untreated or stimulated with fMLP served as negative and positive (+fMLP) controls. Lysates were blotted for (A,D) Akt phosphorylation at Thr308. HRP was inactivated followed by reblotting of each membrane with antibodies toward total Akt and β-actin antibody as a loading control. Membranes were imaged for (B,E) total Akt and (C,F) β-actin separately.

**Supplementary Figure 5: Enlarged images of actin florescent microscopy presented in Figure 6.** Murine bone marrow neutrophils were isolated and **(A)** treated with 100 nM of MspA or MspTL for 30 min, then stimulated with 1 μM fMLP for 1 min at RT or **(B)** Treated with 30 μM ISA-2011B for 1 hr at 37C followed by 100nM MspA or MspTL for 30 min at RT. Cells were fixed to coverslips and stained for F-actin by phalloidin.
